# Riboflavin in neurological diseases: therapeutic advances, metabolic insights, and emerging genetic strategies

**DOI:** 10.3389/fneur.2025.1663136

**Published:** 2025-09-02

**Authors:** Zhiming Tao, Jinglu Huo, Xiaosheng Hao, Jianmin Liang

**Affiliations:** ^1^Department of Pediatric Neurology, Children’s Medical Center, The First Hospital of Jilin University, Changchun, China; ^2^Jilin Provincial Key Laboratory of Pediatric Neurology, Changchun, China; ^3^Neuromedical Center, First Hospital of Jilin University, Changchun, China

**Keywords:** riboflavin, vitamin B2, neurological diseases, mitochondrial dysfunction, gene therapy, combination therapy

## Abstract

**Background:**

Riboflavin (vitamin B2), a precursor of flavin mononucleotide (FMN) and flavin adenine dinucleotide (FAD), is essential for mitochondrial function, redox balance, and neuronal viability. Impairments in riboflavin transport and metabolism contribute to a growing spectrum of neurological diseases.

**Objective:**

This review provides a comprehensive update on the therapeutic applications, metabolic mechanisms, and gene-based strategies involving riboflavin in neurological disorders.

**Methods:**

We systematically analyzed clinical and experimental studies published between 2012 and 2025, focusing on riboflavin-responsive conditions and molecular mechanisms relevant to neurological pathology.

**Results:**

Riboflavin supplementation—particularly in high doses—has demonstrated substantial efficacy in conditions such as riboflavin transporter deficiency (RTD), multiple acyl-CoA dehydrogenase deficiency (MADD), and migraine. Emerging data suggest potential benefit in Parkinson’s disease, Alzheimer’s disease, multiple sclerosis, and acute brain injury. Mechanistically, riboflavin supports mitochondrial bioenergetics, antioxidant systems, and epigenetic regulation. Recent advances in gene therapy and pharmacological chaperones targeting riboflavin-dependent pathways offer promising therapeutic directions.

**Conclusion:**

Riboflavin is evolving from a conventional micronutrient into a multifaceted therapeutic agent in neurology. Integration of gene-based approaches, targeted delivery systems, and biomarker-guided interventions may establish riboflavin as a key component of precision medicine strategies for neurological disorders.

## 1 Introduction

Riboflavin (vitamin B2) is a water-soluble micronutrient essential for cellular energy metabolism, antioxidant defense, and neuronal function (1, 2). As the precursor of flavin mononucleotide (FMN) and flavin adenine dinucleotide (FAD), riboflavin is indispensable for redox reactions, mitochondrial respiration, and maintenance of myelin integrity (3).

Over the past decade, increasing evidence has revealed that disturbances in riboflavin metabolism, transport, or utilization contribute to a spectrum of neurological disorders. These include primary mitochondrial diseases, multiple acyl-CoA dehydrogenase deficiency (MADD), Brown–Vialetto–Van Laere syndrome (BVVL), as well as more prevalent conditions such as migraine, Parkinson’s disease, multiple sclerosis, and cognitive impairment (4–8).

While several studies and reviews have addressed the therapeutic role of riboflavin in specific diseases, few have integrated the metabolic, genetic, and therapeutic aspects of riboflavin across the neurological disease continuum. In particular, recent advances in gene therapy, riboflavin transporter biology, and flavoenzyme-targeted pharmacology remain underrepresented in the literature (9–11).

Here we focus on studies published from 2012 to 2025 to systematically review the role of riboflavin in neurological diseases by summarizing clinical and preclinical evidence, exploring underlying mechanisms, and highlighting emerging therapeutic strategies—including gene-based and combination metabolic therapies. We hope to provide a comprehensive update and perspective for clinicians and researchers in neurology, metabolism, and neuropharmacology.

## 2 Literature search strategy

We conducted a comprehensive literature search to identify relevant studies on the role of riboflavin in neurological diseases. The search was performed in the PubMed, Web of Science, Embase, and Scopus databases for articles published from January 1, 2012, to May 31, 2025. Search terms included combinations of the following Medical Subject Headings (MeSH) and keywords: “riboflavin” OR “vitamin B2” OR “flavin adenine dinucleotide” OR “flavin mononucleotide” AND “neurological disease” OR “nervous system disorder” OR “brain disease” AND “mitochondria” OR “oxidative stress” OR “inflammation” OR “gene therapy” OR “riboflavin transporter deficiency” OR disease-specific terms (e.g., “migraine,” “Parkinson’s disease,” “multiple sclerosis,” “MADD,” “BVVL”).

We included original research articles, randomized controlled trials, meta-analyses, systematic reviews, and high-quality narrative reviews published in peer-reviewed journals. Inclusion was restricted to English-language publications involving human participants, animal models, or cellular systems with relevance to neurological outcomes.

Exclusion criteria included conference abstracts, editorials, non-English papers, and studies unrelated to the nervous system or lacking mechanistic or therapeutic focus.

The final set of references was curated by two independent reviewers. Key findings were categorized according to disease type, mechanistic pathway, and level of evidence, following PRISMA and SANRA guidance for narrative reviews.

## 3 Riboflavin and neurological disorders

In the sections that follow, each disease is introduced through its mechanistic link to riboflavin metabolism, then examined in light of pre-clinical data, clinical experience, and expert-consensus recommendations. The narrative closes with a brief appraisal of unmet needs and translational prospects, underscoring how precision dosing, biomarker-guided stratification, and next-generation delivery systems could consolidate riboflavin’s place in neurotherapeutics. To orient the readers, we synthesis the key disorders covered, highlighting core mechanisms, dosing ranges, therapeutic responses, and evidence strength (Table 1).

**Table 1:** Riboflavin-responsive neurological disorders: mechanisms, dosing, outcomes, and evidence strength.

Disorder	Mechanistic link to riboflavin	Typical effective dose	Therapeutic response	Evidence strength
Riboflavin transporter deficiency (RTD)	*SLC52A2/A3* loss → FMN/FAD depletion, mitochondrial failure	10–60 mg kg^−1^ day^−1^ (oral/IV) (20)	≈70–80% improve or stabilize if treated early	Multicenter observational; Class I guideline
Multiple acyl-CoA dehydrogenase deficiency (MADD)	ETFDH/ETFA/ETFB instability → FAD-dependent β-oxidation collapse	20–60 mg kg^−1^ day^−1^ children; 100–400 mg day^−1^ adults (26, 27)	Rapid reversal of myopathy & metabolic crises	Cohort studies; consensus statement
Migraine	Mitochondrial energy deficit lowers cortical spreading depression threshold	400 mg day^−1^ (31, 32)	≈50% reduction in monthly attacks	Meta-analyzed RCTs; Level B guideline
Parkinson’s disease	Impaired glutathione recycling & complex-I stability; gut riboflavin deficit	≈90 mg day^−1^ (30 mg q8 h) in pilot (7)	Motor score ↑ in small open-label study	Preliminary; need RCT
Alzheimer’s disease	Oxidative stress & Nrf2 pathway dysfunction	Pre-clinical dosing; human dosing under exploration (37)	Memory rescue in APP/PS1 mice; human data mixed	Animal + observational
Multiple sclerosis	Flavoprotein-dependent myelin & anti-inflammatory effects	50 mg riboflavin (Cytoflavin^®^) bid (41)	0.5-point EDSS gain in open-label study	Small trials; low-moderate
L-2-Hydroxyglutaric aciduria	L2HGDH FAD-dependent enzyme loss	100–300 mg day^−1^ (43)	Partial or variable biochemical & clinical response	Case reports
Friedreich’s ataxia	Secondary complex-II & aconitase dysfunction	≈15 mg kg^−1^ day^−1^ (combo regimens) (49)	≈20% ICARS improvement in pilot	Pilot studies
AIFM1-related encephalopathy	FAD-binding protein instability	200–400 mg day^−1^ (55)	ICARS ↓ 20–44% in case series	Case series
Traumatic brain injury (TBI) & ischemic stroke	Oxidative and mitochondrial dysfunction, mitigated by riboflavin’s antioxidant and anti-inflammatory effects	10–50 mg kg^−1^ day^−1^ (pre-clinical IP/oral); human dosing exploratory (57)	infarct volume↓, improved neuro-behavioral scores in rodents; limited early-phase human data	Pre-clinical; pilot clinical

BBB, blood–brain barrier; EDSS, Expanded Disability Status Scale; ICARS, International Cooperative Ataxia Rating Scale; IP, intraperitoneal; RCT, randomized controlled trial. Evidence strength adapted from Oxford Centre for Evidence-Based Medicine levels.

### 3.1 Riboflavin transporter deficiency

Riboflavin transporter deficiency (RTD)—historically termed Brown–Vialetto–Van Laere or Fazio-Londe syndrome—is a rare, early-onset motor neuron disease that couples the biochemistry of riboflavin with overt neurodegeneration (12). Pathogenic loss-of-function variants in *SLC52A2* or *SLC52A3* abrogate membrane uptake of riboflavin, precipitating secondary FMN/FAD depletion in metabolically demanding neurons and glia (13, 14). Consequent mitochondrial energy failure drives axonal degeneration, sensorineural deafness and progressive bulbar weakness—the cardinal clinical trial of RTD (15, 16).

Mechanistic work in patient fibroblasts and iPSC-derived motor neurons confirms that transporter mutations collapse intracellular FMN/FAD pools, impair respiratory-chain flux and trigger neurite loss; high-dose riboflavin (≥10 mg kg^−1^ day^−1^) or flavin esters rapidly restore ATP production and cell viability *in vitro* (17). Translational concordance is strong: zebrafish *SLC52A3* knock-down and *SLC52A3*^−/−^ mice recapitulate hearing loss, ataxia and early death, all of which improve with systemic riboflavin supplementation (17, 18). Most recently, AAV9-mediated *SLC52A2* gene replacement normalized FMN/FAD levels, rescued spinal motor neurons and reversed motor deficits when delivered pre-symptomatically, underscoring the feasibility of curative therapy (19).

Clinically, oral or intravenous riboflavin remains first-line treatment. Multicenter series indicate that 70–80% of genetically confirmed patients improve or stabilize on 10–60 mg kg^−1^ day^−1^, with greater benefit when therapy starts within 12 months of symptom onset (20). Bulbar and respiratory functions often recover, but established auditory and visual loss are less reversible, emphasizing the need for early recognition (16). Although no randomized trials exist, the uniformity of observational data has led GeneReviews^®^ and European neurometabolic taskforces to issue class I recommendations for immediate high-dose riboflavin upon molecular diagnosis (14).

Looking ahead, riboflavin therapy serves as a life-prolonging bridge rather than a definitive cure in advanced disease. Pipeline strategies include (i) AAV gene replacement for *SLC52A2/A3*, (ii) brain-targeted delivery of active flavins, and (iii) small-molecule chaperones that stabilize misfolded transporters—all of which will require biomarker-guided protocols and functional imaging endpoints to quantify long-term efficacy (19).

### 3.2 Multiple acyl-CoA dehydrogenase deficiency

Multiple acyl-CoA dehydrogenase deficiency (MADD) also known as glutaric academia type II or lipid-storage myopathy—is caused by loss-of-function variants in ETFDH, ETFA, ETFB or, more rarely, FLAD1. These genes encode the electron-transfer flavoprotein system that funnels electrons from dozens of FAD-dependent dehydrogenases into the respiratory chain. Pathogenic variants destabilize the flavoproteins, deplete intracellular flavin pools and precipitate multisystem energy failure. Clinically, “riboflavin-responsive” MADD is distinguished by its dramatic reversal of myopathy, hypoglycemia and liver dysfunction after high-dose riboflavin supplementation.

*In vitro* work using patient fibroblasts and iPSC-derived myoblasts shows that riboflavin repletes FAD, rescues ETF/ETF-QO stability and restores β-oxidation flux; these effects are magnified when combined with carnitine or CoQ10 co-therapy (21, 22). A 2022 cohort of 31 genetically confirmed patients demonstrated ≥2-grade improvement in muscle power within 3 months of 20–60 mg kg^−1^ day^−1^ oral riboflavin, with sustained biochemical normalization at two-year follow-up (23). More recent multicenter data (*n* = 42, 2025) confirm dose–response benefits and highlight early treatment (<6 months from onset) as the strongest predictor of full ambulation (11). Late-onset presentations, including adult hyperammonemia encephalopathy, also respond, though residual axial weakness and peripheral neuropathy are common (24, 25). GeneReviews^®^, Orphanet and the 2025 metabolic-medicine consensus statement all recommend immediate riboflavin (100–400 mg day^−1^ in adults or 10–60 mg kg^−1^ day^−1^ in children) plus carnitine, with dietary fat restriction and avoidance of catabolism as adjunct measures (26, 27).

Although riboflavin therapy markedly improves clinical outcomes in MADD, several challenges remain. The lack of predictive biomarkers limits early identification of responders, and genotype–phenotype correlations are still incomplete. Current newborn screening may miss riboflavin-responsive cases, underscoring the need for expanded panels incorporating genetic or second-tier biochemical markers. Novel strategies—such as flavin analogues, ETF-stabilizing compounds, and AAV-mediated ETFDH gene therapy—are under preclinical development to enhance treatment efficacy and provide long-term correction. Moving forward, precision diagnostics and mechanism-based interventions will be key to maximizing therapeutic benefit in this highly treatable disorder.

### 3.3 Migraine

Migraine is increasingly recognized as a mitochondrial energy-deficient disorder. Impaired oxidative phosphorylation may lower cortical spreading depression (CSD) thresholds and promote the release of calcitonin gene-related peptide (CGRP), both key drivers of migraine attacks. Riboflavin, as a precursor of FMN and FAD, enhances mitochondrial redox capacity and reduces oxidative stress, which may mitigate migraine susceptibility and severity (28).

Experimental studies, including MR spectroscopy, have shown that riboflavin supplementation increases the phosphocreatine-to-ATP ratio and reduces reactive oxygen species in the brain, supporting a neuroprotective effect. A randomized controlled trial involving 55 adult patients demonstrated that 400 mg/day of riboflavin for 3 months reduced monthly migraine frequency by ~50% and headache days by ~40% compared to placebo (*p* < 0.01) (29). Subsequent meta-analyses combining data from four RCTs (*n* ≈ 300) confirmed that 200–400 mg/day significantly decreased headache duration and pain intensity, with a pooled standardized mean difference (SMD) of −0.25 (95% CI: 0.40–0.09). However, low-dose regimens (≤100 mg/day) showed inconsistent efficacy. Pediatric evidence remains limited and inconclusive, possibly due to age-related differences in metabolic demand and riboflavin kinetics (30). Current guidelines from the American Academy of Neurology and the American Headache Society give riboflavin a Level B recommendation for migraine prevention, with a typical dose of 400 mg/day and excellent safety profile (31, 32).

Although riboflavin is a safe and accessible preventive therapy for migraine, several gaps limit its optimal use. Biomarkers that predict individual response are lacking, hindering patient stratification. Pediatric data remain limited, with unclear dosing and inconsistent outcomes, highlighting the need for age-specific studies. The potential for synergistic effects with other metabolic agents—such as coenzyme Q10 or magnesium—warrants further investigation. Advances in sustained-release formulations and gut-brain axis-targeted delivery may enhance efficacy and adherence. Future efforts should focus on refining patient selection and integrating riboflavin into personalized preventive strategies.

### 3.4 Parkinson’s disease

Parkinson’s disease (PD) is increasingly understood as a systemic metabolic disorder with neurodegenerative consequences. Recent studies have discovered reduced gut bacterial synthesis of riboflavin, which may contribute to neuroinflammation and intestinal permeability—two processes implicated in α-synuclein pathology and dopaminergic cell loss (33). Meanwhile, riboflavin influences glutathione reductase activity, mitochondrial complex integrity, and iron homeostasis, all critical in PD pathogenesis (7).

At the mechanistic level, cell and animal studies suggest riboflavin deficiency impairs glutathione recycling and triggers accumulation of reactive oxygen species and mitochondrial DNA damage—hallmarks of PD pathology (34). A small open-label intervention (*n* = 19) combining riboflavin (30 mg every 8 h) with a red-meat-free diet reported a mean motor function improvement from 44 to 71% over 6 months (*p* < 0.001), with no serious side effects (7). The recent meta-analysis of microbiome data across five countries confirms diminished riboflavin-related bacterial genes in PD, and highlights its association with reduced short-chain fatty acids and polyamines—molecules linked to gut barrier integrity and neuroinflammation (35). However, standardized clinical trials are still lacking, and professional guidelines do not yet list riboflavin among established PD treatments.

Future investigation should emphasize well-powered, placebo-controlled trials assessing the efficacy of riboflavin, alone or with biotin, in slowing PD progression, with motor and non-motor outcomes. Integration of microbiome profiling may help identify subgroups likely to benefit from supplementation. Further mechanistic studies are needed to clarify how riboflavin modulates glutathione metabolism, mitochondrial stability, α-synuclein aggregation, and iron regulation. Finally, innovations in delivery—like gut-targeted or sustained-release formulations—may improve compliance and biological effectiveness, bridging the transition from metabolic adjunct therapy to a precision neuroprotective strategy in PD.

### 3.5 Alzheimer’s disease

Alzheimer’s disease is characterized by oxidative stress, mitochondrial dysfunction, amyloid-β/tau accumulation, and neuroinflammation. Riboflavin, as the precursor of FAD and FMN, supports antioxidant defense (e.g., glutathione reductase, catalase, SOD) and mitochondrial energy production—both compromised in AD. Animal studies in APP/PS1 transgenic mice show that riboflavin reverses memory deficits, reduces ROS/MDA, and activates the Nrf2 antioxidant pathway (36). *In vitro*, FMN modulates microglial TNFR1/NF-κB signaling, offering an additional anti-inflammatory mechanism (10).

Preclinical Alzheimer models treated with riboflavin show enhancement in antioxidant enzyme activity and improved spatial memory performance in tasks like the Morris water maze (36). Population studies also link higher dietary riboflavin intake to better cognitive function and reduced dementia risk in older adults (37). However, randomized clinical trials of broader B-vitamin supplementation (B12, B6, folic acid) have yielded mixed results—some show slowed brain atrophy or homocysteine reduction, but no clear cognitive benefit in established AD (38).

Future research should focus on clinical trials using riboflavin (alone or combined with FMN/FAD) at optimized doses, targeting early and preclinical AD stages. Biomarker-guided inclusion—e.g., homocysteine levels, Nrf2 activity, microglial inflammation markers—may help identify responders. Development of sustained-release or brain-targeted riboflavin formulations could enhance CNS delivery and patient adherence. Integration of riboflavin into multimodal preventive regimens alongside lifestyle and genetic risk stratification offers a promising avenue for neuroprotection in AD.

### 3.6 Multiple sclerosis

Myelin maintenance and axonal energy supply rely on dozens of FAD-dependent flavoproteins that govern β-oxidation, antioxidant recycling, and sphingolipid synthesis. Experimental riboflavin insufficiency produces spinal demyelination and neuro-inflammation reminiscent of multiple sclerosis (MS), whereas repletion restores redox balance and supports remyelination. Thus, adequate riboflavin is biologically plausible as a neuroprotective and fatigue-modulating adjunct in MS.

In the experimental autoimmune encephalomyelitis (EAE) model, oral riboflavin (20 mg/kg/day) ameliorated motor deficits, suppressed reactive oxygen species, and modulated immune signaling by downregulating interleukin-6 (IL-6) and enhancing brain-derived neurotrophic factor (BDNF) expression, reflecting combined antioxidant and anti-inflammatory effects (39, 40). A 2024 open-label study of Cytoflavin^®^ (containing riboflavin 50 mg per dose) in 68 relapsing-remitting MS patients reported a mean 0.5-point improvement in EDSS and faster Symbol Digit Modalities Test times after 12 weeks, without serious adverse events. Earlier small controlled trials summarized in a systematic review found decreased serum anti-myelin antibodies and modest functional gains, but sample sizes were <40 and heterogeneity high (41). Observational cohorts link higher dietary riboflavin intake with lower MS risk and reduced inflammatory diet scores, supporting a preventive signal (40, 42). No major neurology guideline presently recommends riboflavin, but a 2025 metabolic-medicine consensus recognizes it as a low-toxicity option for mitochondrial-targeted supportive care.

Priority areas include placebo-controlled trials that test riboflavin—alone or combined with coenzyme Q10 or l-carnitine—against fatigue, cognitive decline, and neurofilament-light elevation in early MS; biomarker-driven enrolment using oxidative-stress or flavoprotein signatures; and delivery innovations (e.g., sustained-release or brain-penetrant pro-flavins) to maximize central uptake. Clarifying gene–nutrient interactions (e.g., *SLC52A* polymorphisms) may further refine personalized supplementation strategies.

### 3.7 L-2-hydroxyglutaric aciduria

L-2-hydroxyglutaric aciduria (L-2-HGA) is a rare autosomal recessive neurometabolic disorder caused by pathogenic variants in L2HGDH, encoding the mitochondrial FAD-dependent L-2-hydroxyglutarate dehydrogenase. Loss of enzyme activity leads to the accumulation of L-2-hydroxyglutarate, a “metabolic repair” failure that triggers neurotoxicity, demyelination, cerebellar atrophy, seizures, developmental delay, and extrapyramidal symptoms. As the enzyme requires FAD as a cofactor, riboflavin supplementation is a logical therapeutic approach to enhance residual enzymatic activity.

A seminal case report in a 16-year-old patient treated with 100 mg/day riboflavin over nearly 2 years showed partial improvement in cognitive and motor performance. Urinary L-2-hydroxyglutarate levels dropped from 5,990 to 1,490 mmol/mol creatinine. Discontinuation of riboflavin led to reversal of these gains, which returned upon reinitiation. Higher doses (200 mg/day) offered only marginal further benefit, and 300 mg/day provided no additional effect (43). A 2025 pediatric case with L2HGDH mutations treated with 100 mg/day for 6 months showed no detectable clinical or biochemical response, indicating variable sensitivity (44). Broader literature supports that riboflavin, often in combination with l-carnitine, can lower L-2-hydroxyglutarate levels and ameliorate neurological symptoms, though most data are limited to case reports and small series (45).

L-2-HGA exemplifies a disease-specific metabolic target for riboflavin therapy. Future research should focus on identifying predictors of responsiveness—e.g., enzyme activity assays or genotype profiles—since response is inconsistent. Optimal dosing strategies warrant investigation, balancing efficacy and patient tolerance. Randomized controlled trials or registry-based cohorts with systematic phenotyping, neuroimaging, and biochemical monitoring are needed. Finally, combining riboflavin with adjunctive agents like l-carnitine, and exploring direct FAD supplementation, could maximize enzymatic rescue in refractory cases.

### 3.8 Friedreich’s ataxia

Friedreich’s ataxia is an inherited neurodegenerative disorder caused by GAA triplet repeat expansions in the FXN gene, which lead to frataxin deficiency. The loss of frataxin disrupts mitochondrial iron–sulfur-cluster formation, triggers oxidative stress, and negatively impacts FAD-dependent enzymes including mitochondrial complex II and aconitase. Studies have demonstrated that riboflavin or direct FAD supplementation in cellular and animal models can restore these enzyme activities, reduce reactive oxygen species, and improve motor coordination (46).

Cell and mouse models demonstrate that riboflavin or direct FAD repletion normalizes aconitase and complex-II activities, limits lipid peroxidation, and improves motor coordination (47, 48). Clinically, two pilot studies have explored riboflavin based combination regimens: (i) triple therapy with deferiprone + idebenone + riboflavin (15 mg/kg/day) in 10 patients yielded a mean 20% improvement in ICARS scores over 12 months without major safety signals (49); (ii) darbepoetin alfa + idebenone + riboflavin achieved similar functional gains and ferritin normalization in a separate open trial (50). No large, randomized trial of riboflavin monotherapy exists, and current guidelines focus on the FDA-approved nuclear-factor-E2-related-factor-2 activator omaveloxolone (Skyclarys^™^) and emerging AAV-FXN gene therapies such as LX2006 (51, 52).

Future efforts should include placebo-controlled trials assessing riboflavin or FAD as add-on therapies to existing standard-of-care agents, with biomarker-driven enrolment based on serum frataxin, oxidative stress panels, and spinal-cord magnetization-transfer imaging. Development of brain-penetrant flavin prodrugs or sustained-release formulations may improve delivery across the blood–brain barrier. Ultimately, combining riboflavin with iron chelation, antioxidant therapy, and gene replacement could offer a multi-targeted approach to neuroprotection in Friedreich’s ataxia.

### 3.9 AIFM1-related encephalopathy

AIFM1 encodes apoptosis-inducing factor, a mitochondrial FAD-dependent flavoprotein essential for respiratory chain complex biogenesis and caspase independent cell death. Pathogenic AIFM1 variants impair mitochondrial complex I/III/IV assembly, reduce AIFM1 protein levels, and compromise respiratory function. Riboflavin supplementation can partially restore AIFM1 protein expression and enhance mitochondrial respiration in affected cells (53).

Recent case reports have documented early onset motor neuron or encephalopathic phenotypes—such as ataxia, seizures, and neuropathy—in individuals with hemizygous AIFM1 missense variants. *In vitro* studies using patient fibroblasts carrying c.1019T>C (p.Met340Thr) showed that riboflavin deficiency further reduced AIFM1 protein, while high-dose riboflavin partially reversed this loss and improved respiratory chain activity (53). Clinically, two male siblings with progressive ataxia treated with 200 mg daily riboflavin experienced reductions in ICARS scores by approximately 44 and 20% at 6 months, suggesting a positive response despite overall phenotypic variability (54). Moreover, a novel early-onset motor neuron disease phenotype with AIFM1 variant (c.617A>G/p.Gln206Arg) demonstrated clinical stability and improved motor performance over 2 years on 400 mg/day riboflavin (55). Although case numbers remain small and responses are heterogeneous, the accumulated evidence supports riboflavin’s potential to stabilize disease progression in select AIFM1-associated disorders.

The therapeutic potential of riboflavin in AIFM1-related conditions merits rigorous investigation through larger, prospective studies. Key areas include establishing dose–response relationships, defining optimal timing for intervention, and identifying biomarkers (e.g., AIFM1 levels, respiratory chain function, neuroimaging) to predict responder status. Given variability in response, genotype-guided selection may identify subgroups most likely to benefit. Long-term follow-up examining functional scales such as ICARS and survival measures will help validate riboflavin’s role. Ultimately, riboflavin may emerge as a low-risk metabolic adjunct that complements emerging genetic or mitochondrial-targeted therapies in AIFM1 encephalopathy.

### 3.10 Traumatic brain injury and stroke

Traumatic brain injury (TBI) and ischemic stroke are common acute central nervous system injuries that share a characteristic cascade: following the initial mechanical or perfusion insult, mitochondrial dysfunction ensues, leading to excessive reactive oxygen species (ROS) production, calcium overload, and neuronal apoptosis. In recent years, numerous basic studies have highlighted that riboflavin’s metabolic support and anti-apoptotic effects in acute brain injury may represent a novel intervention strategy.

Preclinical studies in rodent models of cortical contusion and ischemia/reperfusion injury consistently demonstrate that riboflavin administration (typically 1–20 mg/kg oral or parenteral) reduces lesion volume, brain edema, astrocytosis, and apoptotic cell death, while enhancing sensorimotor recovery and spatial memory performance (8). One mechanistic study showed that riboflavin can prevent FMN loss from complex I and maintain mitochondrial electron transport during reperfusion. In a recent pediatric TBI model using controlled cortical impact in rats, riboflavin significantly improved neurological severity scores and cognitive recovery, while reducing IL-6, IL-1β, TNF-α, MDA levels and boosting SOD activity, angiogenesis, and vessel density (56). Clinical evidence includes stroke trials where 5 mg/day riboflavin, often in combination with other B vitamins, improved erythrocyte glutathione reductase activity and reduced the proportion of biochemically riboflavin-deficient patients, although clinical outcomes remain understudied (57). These data underpin integrative reviews highlighting riboflavin’s antioxidant, anti-inflammatory, neuroprotective, and neurovascular remodeling effects following cerebral hypoxia/anoxia (8).

Although existing data are promising, rigorous randomized controlled trials are still needed to confirm whether riboflavin—alone or combined with agents like magnesium or glutathione precursors—improves functional outcomes in TBI and stroke. Key priorities include dose–response optimization for acute versus chronic phases, inclusion of biomarkers such as glutathione reductase activity, neuroinflammatory cytokines, and imaging-based lesion metrics, and development of formulations that improve central nervous system bioavailability. Ultimately, a precision medicine approach integrating early biomarker-guided supplementation could position riboflavin as a safe, low-cost therapeutic adjunct in neurorehabilitation protocols for brain injury.

## 4 Metabolic pathways and neuroprotective mechanisms of riboflavin

Riboflavin is not only an essential vitamin for cellular energy metabolism, but also a critical regulator in numerous neurological signaling pathways. Its biological functions are primarily exerted through two active derivatives—FMN and FAD. These cofactors participate in redox reactions, metabolic conversions, and signal transduction by serving as essential components of various flavoproteins. Given the nervous system’s high dependence on continuous energy supply and redox homeostasis, it is exceptionally sensitive to disturbances in riboflavin uptake, metabolism, and utilization.

### 4.1 Biosynthesis and mitochondrial targeting of flavin cofactors

**Figure 1:**
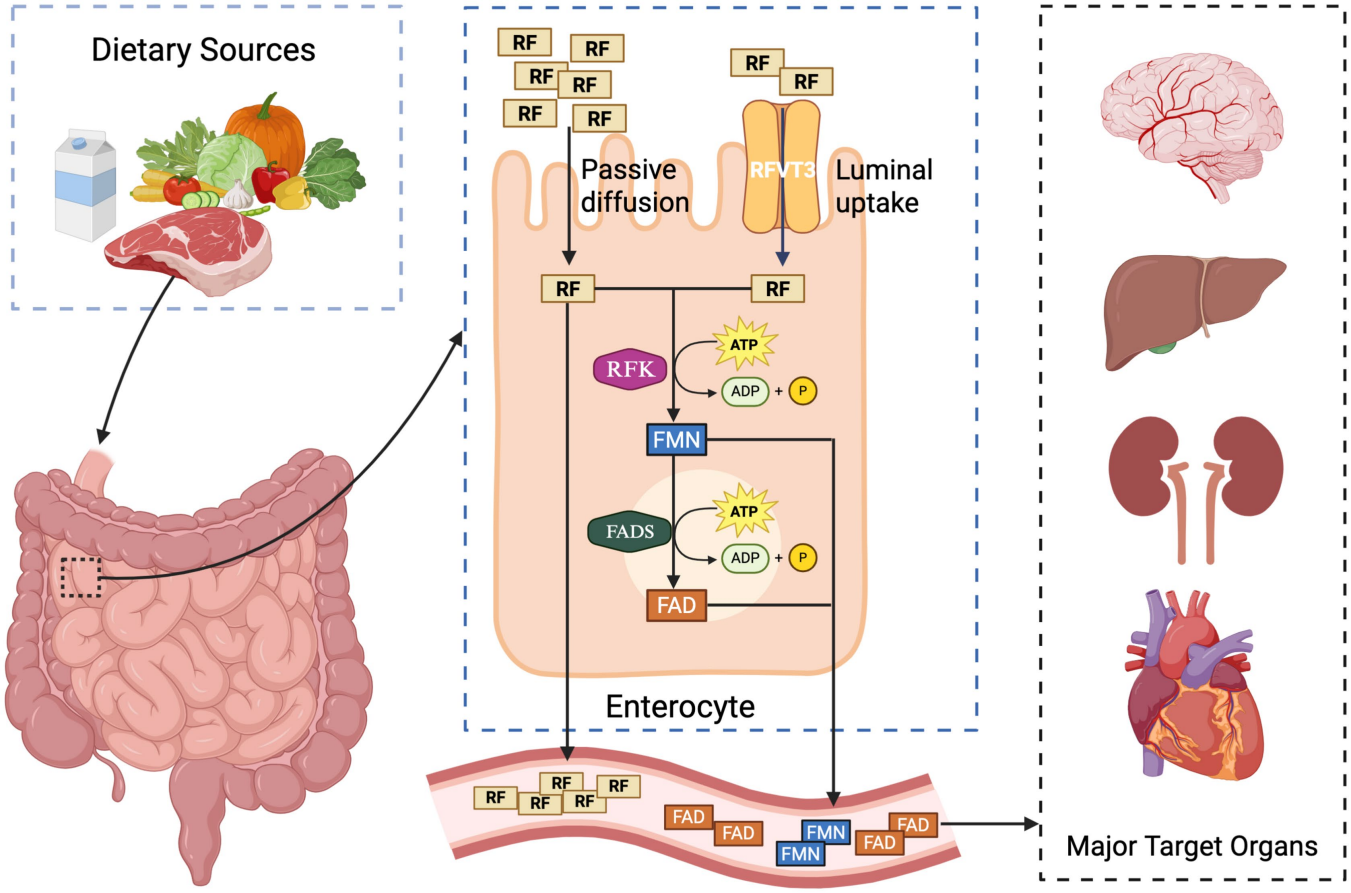
Intestinal absorption, cellular conversion, and systemic distribution of riboflavin (vitamin B2): dietary riboflavin (RF) is absorbed in the small intestine through two main mechanisms: passive diffusion and active transport via the RFVT3 transporter. Once inside enterocytes, RF is sequentially phosphorylated by riboflavin kinase (RFK) to form flavin mononucleotide (FMN), and then adenylated by FAD synthetase (FADS) to generate flavin adenine dinucleotide (FAD), both processes requiring ATP. RF, FMN, and FAD are released into the bloodstream and distributed to metabolically active target organs, including the brain, liver, kidneys, and heart.

Dietary riboflavin is absorbed across the apical membrane of enterocytes by members of the *SLC52* transporter family chiefly *RFVT2/SLC52A2* and *RFVT3/SLC52A3* (58). Inside the cell, riboflavin is phosphorylated by RFK to yield FMN; FMN is subsequently adenylated by FLAD1 to generate FAD (59). Both RFK and FLAD1 reside in the cytosol and within mitochondria, allowing newly formed FAD to accumulate in the mitochondrial matrix, where it serves as an essential co-factor for numerous flavoproteins—including ETF-DH, AIFM1, multiple acyl-CoA dehydrogenases, and glutathione reductase (Figure 1) (60).

**Figure 2:**
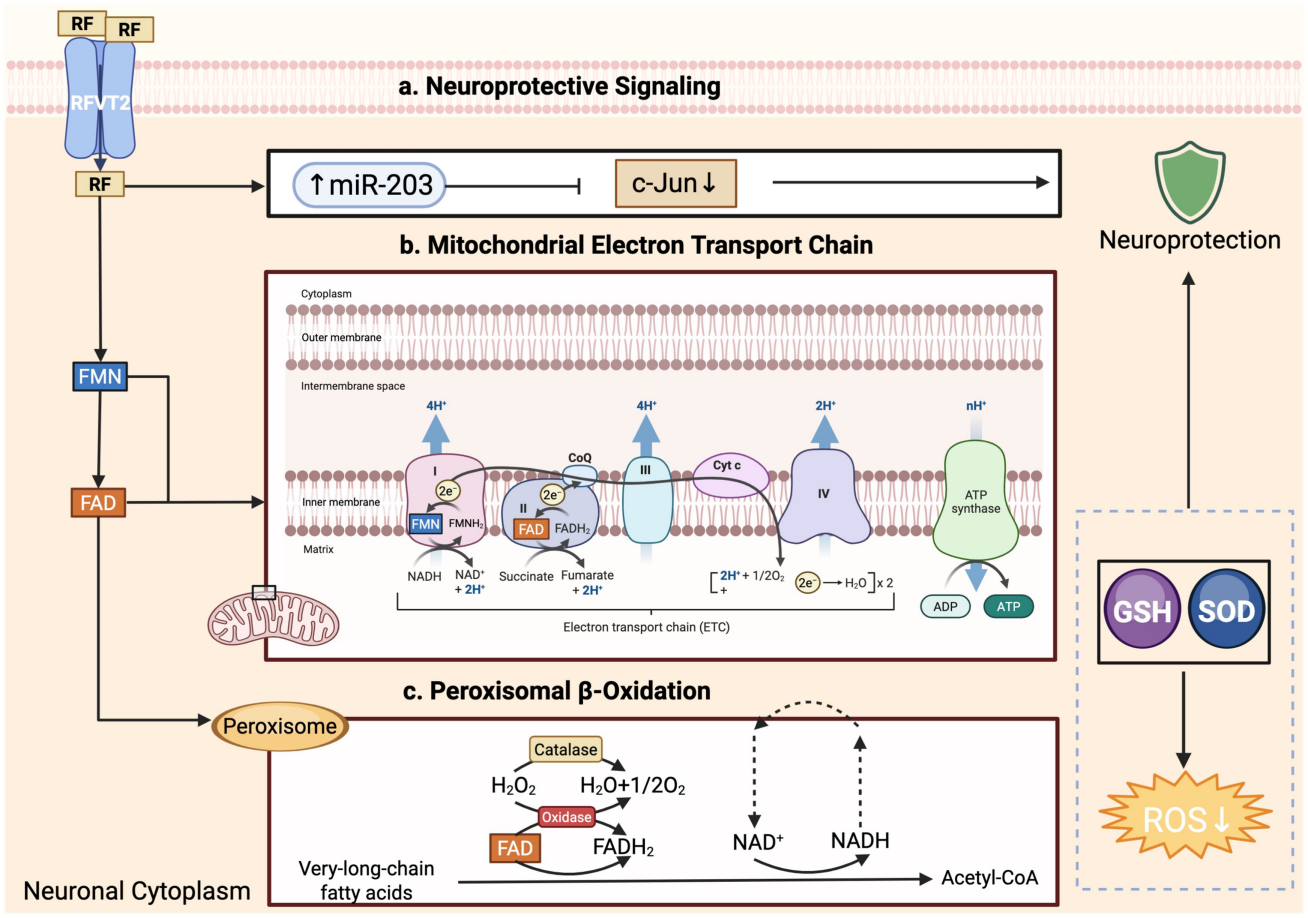
Riboflavin-derived cofactors modulate neuronal bioenergetics, lipid oxidation, and neuroprotective signaling. Riboflavin (RF) enters neurons via RFVT2 transporters and is converted to flavin mononucleotide (FMN) and flavin adenine dinucleotide (FAD) in the cytoplasm. (a) Neuroprotective signaling: RF promotes the expression of miR-203, which suppresses c-Jun activity, thereby enhancing neuroprotection. RF also supports antioxidant defenses by upregulating glutathione (GSH) and superoxide dismutase (SOD), leading to reduced reactive oxygen species (ROS) levels. (b) Mitochondrial electron transport chain (ETC): FMN and FAD serve as electron carriers in complexes I and II, respectively, driving proton translocation across the inner mitochondrial membrane and ATP generation via complex V (ATP synthase). (c) Peroxisomal β-oxidation: FAD-dependent oxidases initiate the breakdown of very-long-chain fatty acids in peroxisomes, producing hydrogen peroxide (H₂O₂), which is detoxified by catalase. This process contributes to cellular redox balance without generating ATP directly. All figures were created by the authors using BioRender.com (license available upon request).

Within neurons, FAD supports the mitochondrial electron-transport chain—most notably complexes I and II—and the α-keto-acid dehydrogenase complexes, processes that are indispensable for maintaining membrane potential and sustaining ATP synthesis (61). FAD also underpins fatty-acid β-oxidation, amino-acid deamination, and purine catabolism, all of which are energetically demanding in the brain (61). Glial cells express a wide array of FAD-dependent enzymes; their metabolic activity modulates the neuro-inflammatory milieu and provides trophic support to neurons (Figure 2) (62, 63). Because of this metabolic integration, any disruption in riboflavin uptake, conversion, or mitochondrial delivery can compromise neuronal survival and glial homeostasis (3, 9).

### 4.2 Regulation of oxidative stress and antioxidant systems

The central nervous system—characterized by high oxygen consumption, intense metabolic activity, and rich lipid content—is highly susceptible to oxidative damage. Riboflavin, via its active cofactor FAD, enhances cellular antioxidant defenses by activating key enzymes such as glutathione reductase, thioredoxin reductase, and flavin-containing monooxygenases. This promotes the glutathione redox cycle, facilitates reactive oxygen species (ROS) scavenging, and suppresses lipid peroxidation markers like malondialdehyde (MDA) and 4-hydroxynonenal (4-HNE). Across multiple experimental models—including Parkinson’s disease, Alzheimer’s disease, and cerebral ischemia-reperfusion injury—riboflavin supplementation has demonstrated consistent neuroprotective effects, reflected in reduced oxidative burden and preservation of neuronal integrity (34).

Mechanistically, riboflavin deficiency leads to diminished glutathione reductase activity, reducing the GSH/GSSG ratio and compromising redox buffering. In Parkinson’s disease models, riboflavin activates the Nrf2/HO-1 pathway, mitigating mitochondrial complex I-mediated ROS overproduction and safeguarding dopaminergic neurons (e.g., substantia nigra) (10, 64). In cerebral ischemia-reperfusion models, riboflavin attenuates NOX2-dependent superoxide generation, lessens cytochrome c release, and inhibits apoptosome activation. In Alzheimer’s disease models, it also upregulates endogenous antioxidative enzymes such as SOD and catalase, mitigating amyloid-β-induced oxidative injury (10).

Riboflavin further modulates redox-sensitive transcription factors including Nrf2 and AP-1, thereby inducing downstream antioxidant genes like NQO1, GCLC, and HO-1. By curbing mitochondrial ROS generation at complexes I and II, riboflavin disrupts the vicious oxidative cycle, leading to diminished lipid peroxidation, preserved mitochondrial integrity, and reduced initiation of cell death pathways—a convergent neuroprotective mechanism across diverse neuropathological contexts (35, 65).

### 4.3 Neuroinflammation and immune signaling interference

Beyond supporting energy metabolism, FAD and FMN critically modulate neuroinflammatory pathways by influencing signaling cascades such as TNFR1/NF-κB, MAPK, and PI3K-Akt. In microglia, FMN suppresses expression of lysine-specific methyltransferase 2B (KMT2B), attenuating TNFR1/NF-κB activation and downstream pro-inflammatory cytokine release (e.g., IL-1β, TNF-α). Appropriate FAD-dependent enzyme activity is likewise essential for blood–brain barrier integrity: deficits in flavin enzyme function compromise endothelial tight junctions, increasing barrier permeability and facilitating neuroinflammatory propagation.

Beyond its metabolic functions, FAD and FMN substantially influence neuroinflammatory signaling, particularly through modulation of pathways such as TNFR1/NF-κB, MAPK, and PI3K-Akt. In microglia, FMN suppresses KMT2B expression, thereby downregulating TNFR1/NF-κB activity and curbing the release of pro-inflammatory cytokines like IL-1β and TNF-α (10, 66). Impaired FAD-dependent enzyme activity is also detrimental to blood–brain barrier (BBB) integrity, leading to downregulation of tight-junction proteins such as claudin-5 and occluding, increased permeability, and enhanced peripheral immune infiltration.

Emerging evidence indicates that riboflavin deficiency skews microglia toward a pro-inflammatory M1 phenotype, characterized by elevated iNOS, IL-1β, and TNF-α, whereas riboflavin supplementation promotes an anti-inflammatory M2 phenotype, with higher IL-10 and TGF-β expression via STAT6 activation (67, 68). In astrocytes, riboflavin modulates connexin-43 expression and enhances glutamate uptake, mitigating excitotoxic and inflammatory cascades. In Alzheimer’s disease models, riboflavin suppresses p38 MAPK and NF-κB activation, reducing amyloid-β-induced neuroinflammation and microgliosis (10).

Together, these findings underscore riboflavin’s dual neuroprotective role: inhibiting pro-inflammatory signaling at the cellular level, and reinforcing BBB stability to reduce neuroinflammatory propagation. These mechanisms help attenuate secondary neuronal injury and foster an environment conducive to repair and resilience.

### 4.4 Epigenetic and protein-stabilization mechanisms

Emerging research reveals that riboflavin, through its FAD cofactor, participates in several epigenetic regulatory mechanisms. FAD is required by α-ketoglutarate-dependent dioxygenases—such as TET DNA demethylases and JmjC family histone demethylases—either directly or indirectly via metabolic interactions (69). In diseases linked to FAD deficiency or disrupted riboflavin metabolism, like L-2-HGA and AIFM1-related encephalopathy, aberrant chromatin states and DNA methylation patterns have been observed, underscoring a tight connection between riboflavin availability and gene expression regulation.

Moreover, in flavoproteinopathies such as AIFM1 or FLAD1 mutations, depletion of FAD leads to misfolding and functional loss of affected proteins. Riboflavin supplementation—by increasing intracellular FAD concentration—can restore cofactor binding, improve protein folding and subcellular localization, and rescue enzymatic activity, an effect known as the flavin chaperoning or chemical chaperone mechanism (58). This protein-stabilizing action has been demonstrated across various riboflavin-responsive metabolic disorders, highlighting its dual role in both metabolic support and proteostasis maintenance.

## 5 Riboflavin in gene therapy and molecular targeting of neurological disorders

With the advent of high-throughput sequencing and broader genetic testing, an increasing number of inherited neurometabolic disorders linked to riboflavin metabolism have been identified. This breakthrough has accelerated research into riboflavin’s role not only as a conventional dietary supplement to correct metabolic deficiencies, but also as a component of gene therapy approaches and enzyme-targeted precision medicine. In disorders such as RTD, MADD, and AIFM1-related encephalopathy, riboflavin supplementation—whether alone or in combination with genetic or pharmacological modulation—has shown promise in stabilizing disease progression and restoring enzyme function. These insights have laid the groundwork for novel therapeutic paradigms where riboflavin acts synergistically with genetic correction or as a pharmacological cofactor to enhance the folding, stability, and activity of mutant flavoproteins.

### 5.1 Genetic mechanisms of riboflavin transport and utilization defects

Dietary riboflavin bioavailability depends on coordinated processes including intestinal absorption, cellular uptake, intracellular activation, and mitochondrial transport. Mutations in critical transporter and enzyme genes disrupt these steps and underlie a spectrum of neurological disorders collectively termed “riboflavin-responsive neurometabolic diseases.” A representative example is riboflavin transporter deficiency (RTD), caused by loss-of-function mutations in *SLC52A2* and *SLC52A3*, which encode the riboflavin transporters RFVT2 and RFVT3, respectively. Such mutations impair cellular uptake of riboflavin, leading to reduced FMN and FAD synthesis and consequent dysfunction of mitochondrial FAD-dependent enzymes, culminating in axonal degeneration, motor neuron disease, and sensory neuropathy. RTD is among the most riboflavin-responsive genetic neurological disorders, with early diagnosis and high-dose supplementation (10–50 mg/kg/day) capable of reversing neurological deficits (26, 27).

Downstream, RFK (riboflavin kinase) is essential for converting riboflavin to FMN. Though pathogenic RFK mutations are rare, animal models have shown that RFK loss results in impaired mitochondrial electron transport, NF-κB activation, cognitive decline, and neuroinflammation, highlighting its disease relevance. FMN also modulates epigenetic regulators like the histone methyltransferase KMT2B, indirectly influencing immune gene expression and neuroimmune balance, emphasizing the broader regulatory role of the RFK pathway.

Further downstream, FLAD1 (FAD synthase) catalyzes FMN conversion to FAD. FLAD1 loss-of-function results in early-onset mitochondrial dysfunction, myopathy, and metabolic acidosis—phenotypes characteristic of “FAD deficiency.” However, many patients retain partial enzyme activity and show clinical response to riboflavin. High-dose supplementation can enhance folding and stability of mutant FLAD1, restoring partial enzymatic function via a biochemical “flavin chaperone” effect (60).

Other FAD-binding flavoproteins, such as AIFM1, are also implicated. AIFM1 mutations disrupt FAD binding, resulting in protein instability, mitochondrial dysfunction, and neurodegenerative phenotypes including brainstem atrophy and motor impairment. High-dose riboflavin has shown efficacy in stabilizing mutant AIFM1 and improving clinical features in some patients, marking it as a prototypical FAD-sensitive genotype.

In addition, L2HGDH, encoding the FAD-dependent L-2-hydroxyglutarate dehydrogenase, is mutated in L-2-hydroxyglutaric aciduria. While evidence for riboflavin supplementation is limited, the theoretical rationale rests on replenishing FAD pools to normalize metabolic flux and mitigate disease progression.

Collectively, these transporters and enzymes—encompassing *SLC52A2/A3*, RFK, FLAD1, AIFM1, and L2HGDH—form the molecular substrate of Table 2. Understanding their mutational impact aids in identifying intervention targets and implementing genotype-informed riboflavin therapies. As structural biology and precision modelling evolve, combined gene/metabolic strategies may enable more effective treatments.

**Table 2:** Genetic defects in the riboflavin pathway and their clinical implications.

Gene	Protein/function	Associated disorder(s)	Inheritance	Riboflavin responsiveness	Additional/emerging therapy
SLC52A2 (RFVT2)	Basolateral riboflavin transporter (neuronal)	RTD type 2	Autosomal recessive	High (life-saving) (4)	AAV9 gene replacement (pre-clinical)
SLC52A3 (RFVT3)	Intestinal/placental riboflavin transporter	RTD type 3	Autosomal recessive	High (4)	AAV-gut targeted vectors
RFK	Riboflavin kinase → FMN	FMN-deficiency syndrome (rare)	Autosomal recessive	Potential (pre-clinical)	mRNA replacement
FLAD1	FAD synthase → FAD	FAD-deficiency myopathy	Autosomal recessive	Partial	CRISPR/Cas9, mRNA therapy
ETFDH/ETFA/ETFB	Electron-transfer flavoprotein system	Riboflavin-responsive MADD	Autosomal recessive	High (26, 27)	Flavin chaperones (lumichrome)
AIFM1	Mitochondrial apoptosis-inducing factor	AIFM1 encephalopathy/axonopathy	X-linked	Variable (53)	AAV + high-dose riboflavin
L2HGDH	L-2-Hydroxyglutarate dehydrogenase	L-2-HGA	Autosomal recessive	Variable (43)	Adjunct l-carnitine, FAD supplementation
FXN (contextual)	Frataxin (iron–sulfur cluster)	Friedreich’s ataxia	Autosomal recessive	Adjunctive (51, 52)	AAV-FXN, omaveloxolone

AAV, adeno-associated virus; MADD, multiple acyl-CoA dehydrogenase deficiency; L-2-HGA, L-2-hydroxyglutaric aciduria. Riboflavin responsiveness: High = >60% clinical improvement; partial = 20–60%; variable = isolated case reports.

### 5.2 Flavoprotein functional defect-related mutation mechanisms

In addition to genes directly involved in riboflavin uptake and metabolism, numerous inherited disorders arise from structural or functional defects in flavoproteins themselves. In these conditions, riboflavin absorption and conversion processes are intact, but mutations in FAD- or FMN-dependent enzymes impair their activity or stability. This, in turn, leads to mitochondrial dysfunction, disrupted metabolism, or energy depletion. Interestingly, some mutant enzymes regain conformational stability in the presence of high cofactor levels, making riboflavin a viable “functional rescue” strategy.

A prime example is multiple acyl-CoA dehydrogenase deficiency (MADD), caused by mutations in ETFDH, ETFA, or ETFB genes encoding electron transfer flavoprotein or its dehydrogenase. These enzymes are strongly FAD-dependent, and mutations often destabilize FAD-binding motifs or enzyme complexes, impairing fatty acid β-oxidation and energy homeostasis. Clinically, many late-onset MADD patients respond dramatically to high-dose riboflavin (100–400 mg/day), confirming residual enzyme function amenable to cofactor restoration—classifying MADD as a prototype FAD-responsive metabolic myopathy (70).

Another mechanism involves flavoproteins whose stability relies on FAD mediated folding, such as AIFM1. In AIFM1-associated encephalopathies, mutations in the FAD-binding domain lead to protein instability, misvocalization, and mitochondrial dysfunction. Supplementation with riboflavin can act as a molecular chaperone, restoring structure and improving function in FAD-domain mutants (71). Likewise, in FLAD1-related mitochondrial diseases, although the enzyme that synthesizes FAD is impaired, high-dose riboflavin may saturate the residual activity and promote correct folding, bypassing the synthesis bottleneck.

Moreover, in conditions not directly caused by flavoprotein gene mutations—such as L-2-hydroxyglutaric aciduria (L2HGA) or Friedreich’s ataxia (FRDA)—metabolic pathology often involves secondary dysfunction of FAD-dependent enzymes and redox disruption. Here riboflavin supports overall enzymatic capacity and mitigates downstream imbalances by enhancing cofactor availability.

Functional FAD depletion without structural mutations has also been reported in other mitochondrial disorders. This “cofactor insufficiency phenotype” suggests that even intact flavoproteins may lose activity when the flavin supply is compromised, expanding the scope of riboflavin-responding conditions.

Ultimately, flavoprotein mutation mechanisms fall into three categories: (1) direct impairment of FAD-binding sites; (2) enzyme unfolding due to cofactor instability; and (3) secondary inactivation through FAD depletion. Recognizing these patterns informs targeted riboflavin-response screening. Advances in structural biology and predictive modelling of flavoprotein-cofactor interactions promise to refine genetic diagnostics and pave the way for genotype-guided, riboflavin-based precision therapies.

### 5.3 Frontiers in gene-centric therapy for riboflavin pathway disorders

With the rapid advancement of gene editing, viral vectors, and mRNA-based therapeutics, targeted correction of key genes in the riboflavin pathway is breaking through the limitations of traditional vitamin supplementation, offering a transformative approach for “riboflavin-responsive” neurometabolic disorders. Most prominently, studies on RTD caused by *SLC52A2/SLC52A3* mutations show that systemic delivery of *AAV9-SLC52A2* in loss-of-function mouse models restores transporter expression, corrects spinal motor neuron energy deficits, reverses motor dysfunction, and extends survival. In parallel, intestine-specific promoters driving *SLC52A3* delivery are being explored to enhance the gut–brain riboflavin axis, reducing peripheral vector load while improving central uptake efficiency (19).

For deeper defects in flavin cofactor synthases such as FLAD1 and RFK, CRISPR/Cas9 correction and mRNA replacement have restored FAD production, mitochondrial membrane potential, and ROS control in patient-derived iPSC models, demonstrating translational promise (72–74). Given that some mutant enzymes retain low-level FAD-binding capacity, a “gene + high-dose riboflavin” synergy strategy has been proposed: in AIFM1 encephalopathies, low-level AAV transduction re-establishes baseline protein expression, while daily riboflavin doses above 400 mg act as a molecular chaperone to stabilize FAD binding a regimen shown to improve ICARS scores and neuroimaging outcomes (54, 75).

Simultaneously, small molecule flavin cofactors have been identified as pharmacological chaperones for patients unsuitable for viral therapy. High-throughput screening in MADD cell lines has identified several lumichrome derivatives that restore mutant ETFDH activity at micromolar concentrations, validating the concept of “pharmacological riboflavin agonists” (76). These compounds avoid the need for permanent genetic modification and offer flexible dosing, making them a valuable complement to gene therapy.

Importantly, secondary FAD depletion has been observed in diseases without primary flavoprotein mutations—such as L-2-hydroxyglutaric aciduria and Friedreich’s ataxia. *In vitro* and worm models show that supplementation with FAD or riboflavin alleviates mitochondrial respiratory deficits and oxidative stress, suggesting cofactor repletion could enhance emerging gene-targeted therapies (46, 74). This finding broadens the therapeutic scope of precision riboflavin interventions, offering new strategies for complex neurometabolic disorders.

Looking ahead, improving tissue- or cell-specific vector targeting, building high-throughput screening platforms for FAD-sensitive variants, and using patient-derived iPSC neurons for personalized preclinical testing will be pivotal in transitioning from experimental use to clinical implementation of integrated “nutrient plus gene-editing” therapies. With advances in protein–cofactor interaction kinetics and structural prediction algorithms, gene therapies targeting riboflavin pathways are poised to move from proof-of-concept toward precision clinical practice, offering hope for patients with refractory neurometabolic diseases.

## 6 Clinical research and future perspectives

### 6.1 Expansion trends in clinical indications

At present, the most established clinical applications of riboflavin are in FAD responsive metabolic disorders such as RTD and MADD. Numerous studies have confirmed that early diagnosis and high-dose riboflavin supplementation can partially reverse neurological injury and even slow disease progression. However, most of this evidence comes from case reports and small observational cohorts, with a conspicuous lack of large-scale prospective trials. Meanwhile, use in more common neurological conditions—such as migraine and Parkinson’s disease—has been driven largely by empirical efficacy and mechanistic inference, with standardized outcome metrics and controlled trials still lacking (3).

Importantly, patient responses to riboflavin vary significantly across diseases, indicative of mechanistic specificity underpinning therapeutic benefit. Identical dosages may yield markedly different physiological effects depending on genotype or pathological context, highlighting the urgent need for stratified approaches and predictive response evaluation systems.

### 6.2 Metabolic combinatorial therapy

Riboflavin’s emergence as the hub of multi-target “metabolic combinatorial therapy” reflects a shift from single-nutrient supplementation toward network-level correction of mitochondrial and redox failure. In classical FAD-responsive disorders such as MADD, high-dose riboflavin given together with carnitine, CoQ10 or α-lipoic acid augments β-oxidation, rescues exercise capacity and improves acyl-carnitine profiles, outcomes repeatedly documented in small series and case-controlled exercise studies (77, 78). A similar rationale underpins regimens for Friedreich’s ataxia, where riboflavin plus antioxidants (CoQ10, α-lipoic acid) counter mitochondrial iron–sulfur-cluster loss and free-radical injury, offering symptom relief beyond monotherapy (79, 80).

In migraine, the best-studied common neurological indication, riboflavin (400 mg/day) paired with CoQ10 (300 mg/day) and magnesium has shown additive reductions in attack frequency and headache days in double-blind trials, a benefit attributed to convergent support of complex-I electron flow, ATP replenishment and cortical excitability control (81, 82). Practical guidance from both specialist societies and lay resources now lists this triple-nutrient combination as first-line nutraceutical prophylaxis (83).

For chronic inflammatory and degenerative conditions—including multiple sclerosis and Parkinson’s disease—compound preparations such as Cytoflavin^®^ (FAD + succinate + niacinamide + L-carnitine) are being evaluated. Pilot RCTs in relapsing MS report modest EDSS improvement and lower corticosteroid need, suggesting that simultaneous enhancement of oxidative phosphorylation, NAD^+^ cycling and membrane repair may decelerate disability accrual (41, 84). Although Parkinson’s data remain heterogeneous, small controlled studies indicate that multi-vitamin protocols combining riboflavin with other mitochondrial cofactors can dampen oxidative stress and improve motor scores, warranting standardized outcome frameworks before widespread adoption (6, 84).

Interventional diversity is accelerating riboflavin is now paired experimentally with NAD^+^ precursors, B-vitamin complexes, and magnesium in an attempt to exploit pathway complementarity—riboflavin for FAD-linked enzymes, CoQ10 for electron transfer, L-carnitine for acyl transport, and magnesium for excitotoxic buffering. Early evidence already suggests that such multiplex regimens outperform single-agent therapy where metabolic heterogeneity blunts isolated responses (81, 85) (Table 3) Future work must dissect mechanism-specific dosing ratios, develop quantitative biomarkers of combinatorial efficacy, and deliver large, stratified, randomized trials to confirm both safety and clinical relevance.

**Table 3:** Emerging riboflavin-based therapeutic platforms and combination strategies.

Approach	Prototype/agent	Target disease(s)	Mechanistic advantage	Development stage
AAV9-SLC52A2 gene replacement (19)	Single-dose systemic vector	RTD	Restores transporter & FMN/FAD pools	Pre-clinical (mouse)
CRISPR/mRNA repair of FLAD1 & RFK (72–74)	Lipid-nanoparticle mRNA	FAD-deficiency myopathy	Restores FAD synthesis	Pre-clinical cell models
Riboflavin-decorated nanoparticles (88)	PEG-PLGA-RF nanocarriers	PD, ischemia, stroke	BBB penetration & sustained release	Animal studies
Pharmacological flavin chaperones (76)	Lumichrome derivatives	MADD (ETFDH)	Stabilize mutant flavoprotein	High-throughput screens
Metabolic combo therapy (49)	Riboflavin + CoQ10 + carnitine	MADD, FA, migraine	Synergistic mitochondrial support	Small clinical trials
Gut-targeted sustained-release riboflavin (35, 62)	Colon-release tablet	PD, migraine	Corrects microbiome-linked deficiency	Early pilot

BBB, blood–brain barrier; CoQ10, coenzyme Q10; FA, Friedreich’s ataxia; PD, Parkinson’s disease; RF, riboflavin. Development stage classification follows FDA guidance: pre-clinical = non-human efficacy; early pilot = human phase 0-I; small clinical trials = phase II.

### 6.3 Smart delivery systems and precision intervention strategies

Recent advances in drug-delivery science are transforming riboflavin from a rapidly cleared, water-soluble vitamin into a precision neuromodulator that can reach therapeutic concentrations within the brain. Liposomal formulations, PEG-PLGA nanoparticles, and dendrimer or polymer micelles have each prolonged plasma half-life and shielded riboflavin from rapid renal loss, while surface ligands—such as wheat-germ agglutinin, transferrin-mimic peptides, and BBB-shuttling sequences—enable active transcytosis across the blood–brain barrier (86–89).

Several groups have reported that riboflavin-decorated nanocarriers not only raise intracerebral FAD/FMN pools but also down-regulate microglial NF-κB signaling and lipid peroxidation in ischemia or Parkinson-like models, thereby uniting anti-inflammatory, anti-apoptotic, and antioxidant benefits in a single platform (10, 90–92).

Looking forward, disease-responsive nanovesicles that release riboflavin in the presence of ROS or acidic pH are being engineered to fine-tune local dosing while minimizing systemic exposure (93).

Parallel to these technological gains, precision-nutrition frameworks are integrating riboflavin delivery with genotype and metabolomic profiling. Variants in *SLC52A2/A3*, FLAD1, and folate-cycle genes alter riboflavin transport or FAD turnover, modulating clinical response; nutrigenomic studies argue that tailoring dose and formulation to such variants can optimize outcomes and reduce pill burden (94, 95). By coupling brain-targeted nanocarriers with patient-specific metabolic fingerprints, a “gene-matched, pathway-matched” strategy is emerging—positioning riboflavin at the center of personalized, systems-level interventions for both rare flavoproteinopathies and common neurodegenerative disorders.

## 7 Conclusion

Riboflavin, long recognized as a cornerstone of cellular redox metabolism, has emerged as a critical modulator of neurological function, extending its role well beyond conventional vitamin physiology. Through its active forms FMN and FAD, riboflavin governs a spectrum of mitochondrial, antioxidant, epigenetic, and neuroimmune pathways, all of which are intimately linked to neural homeostasis and disease pathogenesis. The discovery of riboflavin transporter and flavoprotein gene mutations has not only elucidated the molecular basis of several neurodegenerative disorders but has also enabled effective, often life-altering, treatment with high-dose riboflavin.

Recent advances in riboflavin-responsive conditions—from monogenic syndromes like RTD and MADD to more complex disorders such as Parkinson’s disease and migraine—underscore the therapeutic versatility of riboflavin-based interventions. The integration of gene therapy, metabolic combinatorial strategies, and intelligent delivery systems offers a promising blueprint for precision riboflavin therapeutics. Moreover, biomarker-guided patient stratification, functional imaging endpoints, and iPSC-based modelling will be crucial for translating riboflavin biology into clinical efficacy across broader neurological contexts.

Taken together, riboflavin is no longer merely a metabolic cofactor but a therapeutic nexus at the intersection of nutrition, genetics, and neurology. Future work should focus on defining riboflavin-responsive endophenotypes, validating combination treatment paradigms, and establishing robust clinical frameworks to integrate riboflavin into personalized neurotherapeutic regimens.
